# Overexpression of PRMT6 does not suppress HIV-1 Tat transactivation in cells naturally lacking PRMT6

**DOI:** 10.1186/1743-422X-10-207

**Published:** 2013-06-24

**Authors:** Haran Sivakumaran, Min-Hsuan Lin, Ann Apolloni, Vincent Cutillas, Hongping Jin, Dongsheng Li, Ting Wei, David Harrich

**Affiliations:** 1 Queensland Institute of Medical Research, Molecular Virology Laboratory, 300 Herston Road, Herston, Brisbane 4006, Australia

**Keywords:** HIV, Tat, Protein arginine methyltransferase 6, Protein methylation, A549 cell line

## Abstract

**Background:**

Protein arginine methyltransferase 6 (PRMT6) can methylate the HIV-1 Tat, Rev and nucleocapsid proteins in a manner that diminishes each of their functions in *in vitro* assays, and increases the stability of Tat in human cells. In this study, we explored the relationship between PRMT6 and HIV-1 Tat by determining the domains in each protein required for interaction.

**Methods:**

Through domain mapping and immunoprecipitation experiments, we determined that both the amino and carboxyl termini of PRMT6, and the activation domain within Tat are essential for interaction. Mutation of the basic domain of Tat did not affect the ability of PRMT6 to interact with Tat.

**Results:**

We next used the A549 human alveolar adenocarcinoma cell line, which naturally expresses undetectable levels of PRMT6, as a model for testing the effects of PRMT6 on Tat stability, transactivation, and HIV-1 replication. As previously observed, steady state levels and the protein half-life of Tat were increased by the ectopic expression of PRMT6. However, no down regulation of Tat transactivation function was observed, even with over 300-fold molar excess of PRMT6 plasmid. We also observed no negative effect on HIV-1 infectivity when A549 producer cells overexpressed PRMT6.

**Conclusions:**

We show that PRMT6 requires the activation domain, but surprisingly not the basic domain, of Tat for protein interaction. This interaction between Tat and PRMT6 may impact upon pathogenic effects attributed to Tat during HIV-1 infection other than its function during transactivation.

## Introduction

The HIV-1 Tat protein is a transcriptional activator that plays an essential role during the late phase of viral replication. Its primary function is to significantly enhance proviral gene expression so that HIV-1 RNA and proteins may be produced for later assembly into infectious virion particles. Tat is recruited along with positive transcription elongation factor b (P-TEFb), primarily composed of cyclin T1 and CDK9, to the RNA polymerase II pre-initiation complex (PIC) assembled at the proviral start site of transcription [1]. Localization of Tat and P-TEFb to the PIC is achieved by the association of P-TEFb, initially complexed with a 7SK small nuclear ribonucleoprotein (snRNP) particle [2], with the start site of transcription, and by the coordinated binding of Tat and cyclin T1 to the transactivation response element (TAR) RNA stem-loop structure. TAR is spontaneously formed from the nascent mRNA transcribed by RNA polymerase II during transcriptional initiation. The basic domain of Tat is implicated in binding to TAR, whereas the amino-terminal activation domain of Tat interacts with cyclin T1 [1]. The coordinated binding of Tat and cyclin T1 to TAR enables the release of P-TEFb from the 7SK snRNP particle, the hyperphosphorylation of RNA polymerase II by CDK9, the alleviation of negative elongation factors that arrest the polymerase, and the efficient processivity of transcriptional elongation [3,4].

Tat is posttranslationally modified by host factors shortly before or during transactivation [5]. One class of Tat modification that is of emerging interest is methylation at lysine and arginine residues [6]. Tat is known to be methylated within its basic domain by protein arginine methyltransferase 6 (PRMT6) [7], SETDB1, SETDB2 [8] and Set7/9-KMT7 [9], and can be demethylated by LSD1/KDM1 following methylation of lysine-51 [10]. Most posttranslational modifications of Tat appear to have stimulatory effects on Tat transactivation [11]. In contrast, PRMT6 has been shown to down regulate transactivation by a proposed mechanism in which methylation of Tat arginine-52 interferes with the interaction with TAR [7,12], even though overexpression of PRMT6 results in the increased stability of Tat through inhibition of proteasome-dependent degradation [13]. PRMT6 is a type I enzyme of the protein arginine methyltransferase family [14] which, among other functions, regulates cellular gene expression by methylating arginine-2 of histone H3. Deposition of a methyl group at arginine-2 interferes with the trimethylation of lysine-4 in histone H3, a post-translational modification associated with chromatin remodeling amiable to gene expression [15-18].

In addition to Tat, PRMT6 was also discovered to methylate the HIV-1 Rev [19] and nucleocapsid proteins [20], again within their respective RNA binding domains and in a manner which down regulates function. Overexpression of PRMT6, however, does not affect the stability of Rev [13]. This apparent targeting of three viral proteins by a single host factor has led to the hypothesis that PRMT6 is a HIV-1 restriction factor [7]. In this study, we aimed to firstly explore the relationship between Tat and PRMT6 by mapping the domains of interaction within both proteins, and secondly to develop a robust cellular model for further testing the effects of PRMT6 on HIV-1 replication. We show that the amino and carboxyl termini of PRMT6 are required for interaction and that, unexpectedly, the activation domain but not the basic domain is the site of interaction within Tat. We also demonstrate that the A549 cell line expresses PRMT6 protein at a level undetectable by western blot. Utilizing the A549 cell line in models of Tat transactivation and HIV-1 infectivity, we show that overexpression of PRMT6 does not down regulate either transactivation or infectivity, in contrast to previously published results [7,12]. Our data invite the re-examination of the role of PRMT6 during HIV-1 replication and suggest that further evidence is required before PRMT6 can definitively be considered a restriction factor.

## Results

### The termini of PRMT6 interact with Tat

**Figure 1:**
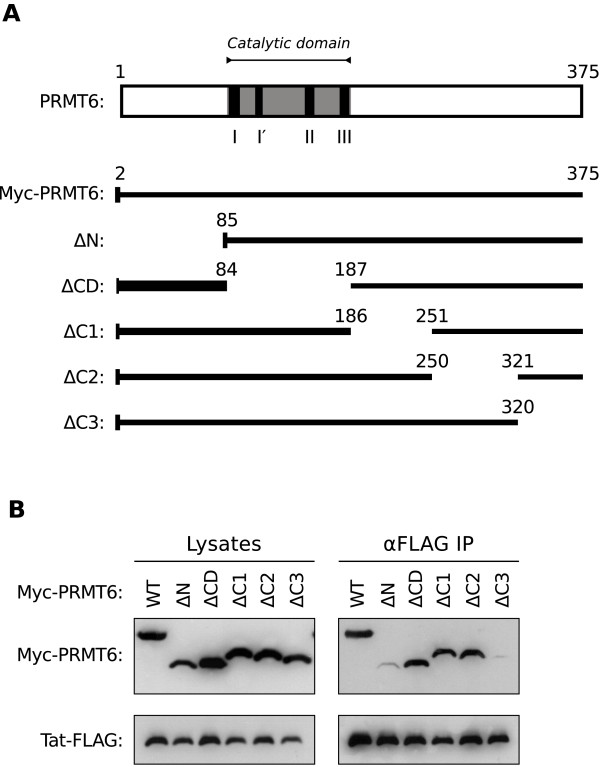
**The termini of PRMT6 are required for the interaction with HIV-1 Tat.** (**A**) *Upper panel*, scale representation of PRMT6 protein showing amino acid positions 1 and 375, the catalytic domain (gray), and the signature methyltransferase motifs (I, I^′^, II and III; black bars). *Lower panel*, scale representations of Myc epitope-tagged PRMT6 (Myc-PRMT6) protein and derivative domain deletion mutants. The numbers above each schema represent the amino acid boundaries of the domain deletions. The amino-terminal Myc epitope tag is represented as a vertical bar. (**B**) Interactions between FLAG epitope-tagged Tat (Tat-FLAG) and wild type or domain deleted Myc-PRMT6 as determined by immunoprecipitation. HeLa cells were transfected to express Tat-FLAG with either wild type Myc-PRMT6 (WT) or a domain deletion mutant of Myc-PRMT6. Immunoprecipitations were performed on lysates prepared from transfected cells using anti-FLAG agarose beads (αFLAG IP). Cell lysates (left panel) and immunoprecipitates (right panel) were western blotted using anti-Myc and anti-FLAG antibodies. Loading of cell lysates was normalized for equal amounts of co expressed *Renilla* luciferase in each sample. The Myc-PRMT6 domain deletion mutant designations are as in **A**. Data are representative of three independent experiments.

Little is known about the functional domains of PRMT6 apart from a catalytic methyltransferase domain that is conserved among all members of the protein arginine methyltransferase family [14,21]. We and others have previously demonstrated that PRMT6 functionally interacts with the HIV-1 Tat protein [7,12,13]. It is not known, however, which region of PRMT6 is required for this interaction. Therefore, to map the region of PRMT6 that interacts with Tat, we created several “domain” deletion mutants in a plasmid expressing MYC epitope tagged-PRMT6 (Myc-PRMT6) in a manner that encompassed the entire length of the PRMT6 protein (Figure 1A). The panel of mutants created included a deletion of the amino terminal region adjacent to the catalytic domain (ΔN), a deletion of the catalytic domain itself (ΔCD), and deletions of three non-overlapping regions at the carboxyl end (ΔC1, ΔC2 and ΔC3; Figure 1A).

To assess the abilities of these mutants to interact with Tat, immunoprecipitations were performed with lysates from HeLa cells co expressing FLAG epitope-tagged Tat (Tat-FLAG) and either wild-type Myc-PRMT6 or one of the domain deletion mutants. Surprisingly, the immunoprecipitation of Tat-FLAG revealed that the catalytic domain mutant of Myc-PRMT6 (ΔCD) was robustly co-immunoprecipitated (Figure 1B, lane ΔCD). This indicates that the catalytic domain, which is responsible for Tat methylation [7], is nonetheless dispensable for the interaction between PRMT6 and Tat. Similarly, the Myc-PRMT6 ΔC1 and ΔC2 mutants interacted with Tat-FLAG to a similar degree to wild-type Myc-PRMT6 (Figure 1B, lanes WT, ΔC1 and ΔC2). In contrast, neither the Myc-PRMT6 ΔN mutant nor the ΔC3 mutant could be efficiently co-immunoprecipitated in repeated experiments (Figure 1B, lanes ΔN and ΔC3), demonstrating that these regions are required for Tat interaction. The data therefore show that the termini of PRMT6, and not its catalytic domain, are the mediators of interaction with HIV-1 Tat.

### The activation domain of Tat interacts with PRMT6

**Figure 2:**
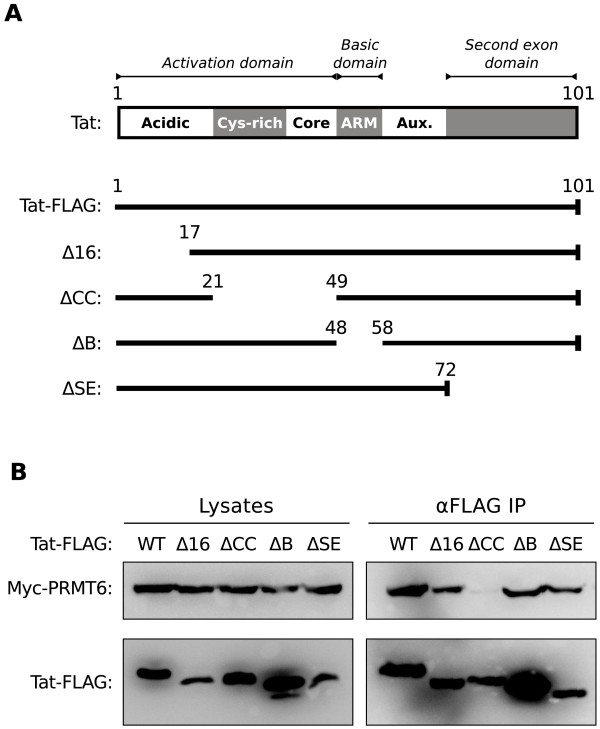
**The activation domain of Tat is required for the interaction with PRMT6.** (**A**) *Upper panel*, scale representation of the two exon-encoded HIV-1 Tat protein showing amino acid positions 1 and 101, functional domains (italicized text) and structural regions (bold text) as defined by [22]. ARM, arginine rich motif; Aux., auxiliary region. *Lower panel*, scale representations of FLAG epitope-tagged Tat (Tat-FLAG) protein and derivative domain deletion mutants. The numbers above each schema represent the amino acid boundaries of the domain deletions. The carboxyl-terminal FLAG epitope tag is represented as a vertical bar. (**B**) Interactions between Myc epitope-tagged PRMT6 (Myc-PRMT6) and wild type or domain deleted Tat-FLAG as determined by immunoprecipitation. HeLa cells were transfected to express Myc-PRMT6 with either wild type Tat-FLAG (WT) or a domain deletion mutant of Tat-FLAG. Immunoprecipitations were performed on lysates prepared from transfected cells using anti-FLAG agarose beads (αFLAG IP). Cell lysates (left panel) and immunoprecipitates (right panel) were western blotted using anti-Myc and anti-FLAG antibodies. Loading of cell lysates was normalized for equal amounts of co expressed *Renilla* luciferase in each sample. The Tat-FLAG domain deletion mutant designations are as in **A**. Data are representative of three independent experiments.

PRMT6 methylates Tat within its basic domain, also known as the arginine rich motif [7,12,13]. To determine if the basic domain is required and sufficient for the interaction between Tat and PRMT6, we similarly created domain deletion mutants in the plasmid expressing Tat-FLAG. Since the functional domains of Tat are well characterized [22], we thus created a panel of mutants (Figure 2A) that included two non-overlapping deletions of the activation domain (Δ16, ΔCC), a deletion of the basic domain (ΔB) and a deletion of the domain encoded by the *tat* second exon (ΔSE).

Immunoprecipitations were perform with lysates from HeLa cells co expressing wild-type Myc-PRMT6 and either wild-type Tat-FLAG or one of the domain deletion mutants. Unexpectedly, immunoprecipitation of the Tat-FLAG basic domain deletion mutant detectably co-immunoprecipitated Myc-PRMT6 (Figure 2B, lane ΔB). The result clearly indicates that the basic domain of Tat is not absolutely critical for the interaction with PRMT6. The Tat-FLAG ΔSE mutant similarly interacted with Myc-PRMT6 (Figure 2B, lane ΔSE). In contrast, deletions made to the activation domain of Tat resulted in somewhat reduced (in the case of the Δ16 mutant) or abrogated (ΔCC mutant) interaction with Myc-PRMT6 (Figure 2B, lanes Δ16 and ΔCC). The data therefore suggest that the Tat activation domain, and not the basic domain, is the important mediator of interaction with PRMT6.

**Figure 3:**
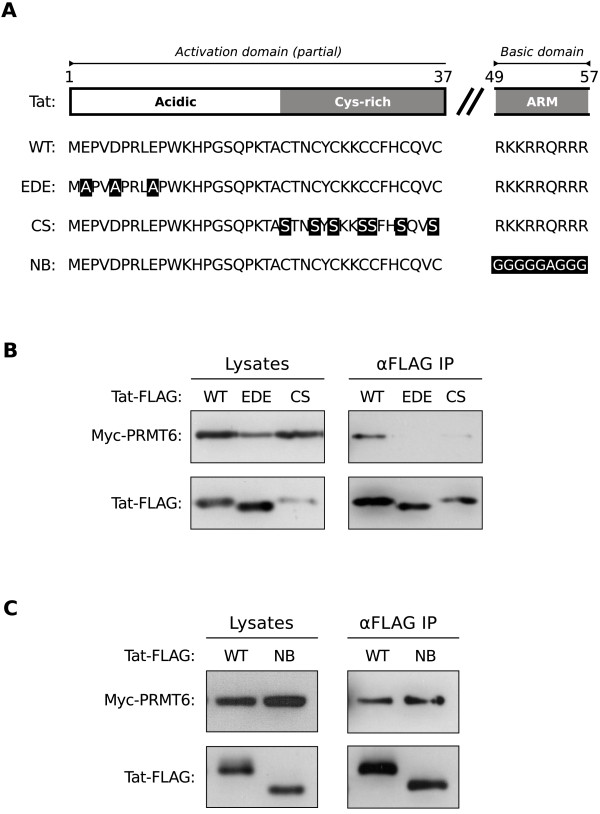
**Acidic and cysteine residues within the Tat activation domain are required for interaction with PRMT6.** (**A**) Schematic representation of the Tat activation domain from amino acids 1 to 37 and the basic domain from amino acids 49 to 57. The wild type (WT) amino acid sequence is shown using the single-letter amino acid code. Sequences of the acidic residues mutant (EDE), the cysteine residues mutant (CS) and the basic domain mutant (NB) are also shown, with substitution mutations indicated in reverse video. ARM, arginine rich motif. (**B**) Interactions between Myc epitope-tagged PRMT6 (Myc-PRMT6) and wild type, EDE mutant or CS mutant FLAG epitope-tagged Tat (Tat-FLAG) as determined by immunoprecipitation. HeLa cells were transfected to express Myc-PRMT6 with either wild type Tat-FLAG (WT) or one of its mutants as indicated. Immunoprecipitations were performed on lysates prepared from transfected cells using anti-FLAG agarose beads (αFLAG IP). Cell lysates (left panel) and immunoprecipitates (right panel) were western blotted using anti-Myc and anti-FLAG antibodies. (**C**) The same experiment as in **B** was performed with the NB mutant of Tat-FLAG. Loading of cell lysates was normalized for equal amounts of co expressed *Renilla* luciferase in each sample. All data are representative of three independent experiments.

To confirm the results revealed by the domain deletion mutants, we repeated the immunoprecipitation experiment with Tat-FLAG mutants in which amino acid substitutions were introduced into the activation and basic domains. Three Tat-FLAG mutants were created (Figure 3A), one in which amino acids Glu-2, Asp-5 and Glu-9 were mutated to alanine residues (EDE mutant), another in which all the cysteine residues between amino acids 21 to 38 were mutated to serine residues (CS mutant), and one in which all the amino acids within the basic domain were mutated to glycine or alanine residues (NB mutant). Immunoprecipitations of the Tat-FLAG mutants in lysates from HeLa cells co expressing Myc-PRMT6 revealed that the EDE mutant could not efficiently interact with Myc-PRMT6 (Figure 3B), whereas the NB mutant remained able to interact (Figure 3C). Expression of the CS mutant in cell lysate samples was less than wild type Tat, so the effect of the cysteine to serine mutations on Tat interaction with PRMT6 remains to be conclusively determined. The amino acids mutated within the activation domain of the EDE mutant have been previously shown to be important for the transactivation function of Tat [23-25]. It is thus demonstrated that key amino acid residues within the activation domain of Tat are necessary not only for transactivation, but also for the interaction between Tat and PRMT6.

### The A549 cell line naturally expresses low levels of PRMT6

Previous investigations of the effects of PRMT6 on HIV-1 replication have involved the use of cell lines that robustly express endogenous levels of PRMT6 protein [12,13,19,20]. Consequently, the degree of influence of this endogenous population of PRMT6 on investigations requiring the transfection of ectopic PRMT6 is unknown. We reasoned that human cell lines that naturally express no or low levels of PRMT6 might make ideal models for exploring the host-pathogen relationship between PRMT6 and HIV-1. To this end, we interrogated the UniGene expressed sequence tag (EST) profile database [26] for candidate tissue types in which PRMT6 ESTs were infrequently reported. When classified by health state, lung tumor and lymphoma tissue were reported to have zero transcripts per million (TPM) of *PRMT6* transcripts, compared to 87 TPM for cervical tumor tissue and 58 TPM for kidney tumor tissue (Table 1). Furthermore, normal lung tissue was reported to express 14 TPM of *PRMT6* transcripts compared to 61 TPM for normal cervical tissue and 47 TPM for normal kidney tissue (Table 1). In contrast, both lung tumor and lymphoma tissue express higher levels of protein arginine methyltransferase 1 (PRMT1), a relatively abundant methyltransferase [14], at 300 and 432 TPM respectively (Table 1).

**Table 1:** **Expressed sequence tag data (shown as transcripts per million) for the *PRMT6 *and *PRMT1 *genes in selected tissue types**

	**Transcripts per million**	
**Tissue**	**PRMT6**	**PRMT1**
*By health state*		
Cervical tumor	87	174
Kidney tumor	58	145
Lung tumor	0	300
Lymphoma	0	432
*By body site*		
Cervix	61	185
Kidney	47	75
Lung	14	179

**Figure 4:**
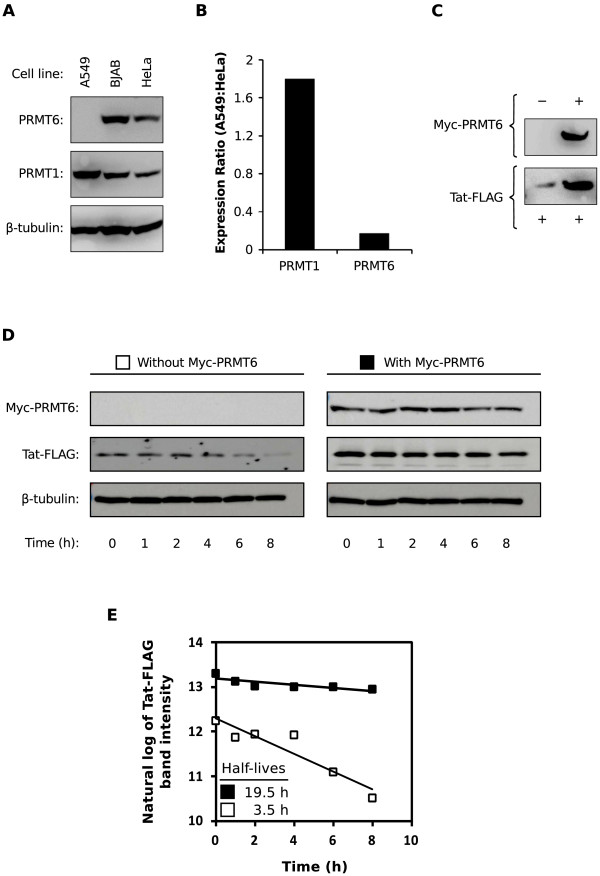
**The A549 cell line does not express detectable levels of PRMT6 protein.** (**A**) Western blot of cell lysates from the A549, BJAB and HeLa cell lines detected with anti-PRMT6, anti-PRMT1 and anti-β-tubulin antibodies as indicated. (**B**) Relative expression ratios of *PRMT1* and *PRMT6* mRNA transcripts in A549 versus HeLa cells. Total RNA were extracted from A549 and HeLa cells before being reverse transcribed into cDNA using random primers. Quantitative PCR was then performed on the cDNA samples using primers specific for *PRMT1*, *PRMT6* and *GAPDH* transcripts. Relative expression ratios for *PRMT1* and *PRMT6* were calculated according to the method of [29]. (**C**) Ectopic Myc-PRMT6 increases the steady state levels of Tat-FLAG protein in A549 cells. Western blotting was performed on A549 cells transfected to express Tat-FLAG with (+) or without (-) Myc-PRMT6. Proteins were detected with anti-FLAG and anti-PRMT6 antibodies, respectively. Loading of cell lysates was normalized for equal amounts of co expressed *Renilla* luciferase in each sample. (**D**) A549 cells transfected to express Tat-FLAG with or without Myc-PRMT6 were treated with cycloheximide and harvested at 0, 1, 2, 4, 6 and 8 h post-treatment. Western blotting was performed on total protein-equalized lysates. β-tubulin levels demonstrate equal sample loadings. (**E**) The Tat-FLAG band intensities in panel **D** were quantified, and their natural log values were plotted as a function of time. Values for Tat-FLAG co expressed with Myc-PRMT6 are indicated by the black boxes and values for Tat-FLAG expressed alone by the white boxes. The calculated Tat-FLAG protein half-lives are shown in the inset.

Guided by the PRMT6 EST data, we therefore obtained human cell lines derived from lung tumor and lymphoma tissue. We chose the A549 human cell line [27], derived from an adenocarcinoma of alveolar basal epithelium origin, and the BJAB human lymphoblastoid cell line [28], derived from an Epstein-Barr virus-negative Burkitt’s lymphoma, respectively. Western blotting for PRMT6 showed that A549 cells had undetectable levels of the protein, in contrast to BJAB and HeLa cells, while all three cell lines had readily detectable PRMT1 protein (Figure 4A). Relative quantification by reverse transcription polymerase chain reaction (PCR) revealed a 5.9-fold deficit of *PRMT6* mRNA transcripts in A549 cells when compared to HeLa cells (Figure 4B). This was determined using Pfaffl’s method of mRNA quantification [29], in which the relative expression ratio of *PRMT6* transcripts between A549 and HeLa cells was normalized to the expression of *GAPDH* transcripts. In contrast, a similar determination of *PRMT1* mRNA levels revealed only a 1.8-fold difference between A549 and HeLa cells (Figure 4B). We therefore demonstrate that the A549 cell line naturally expresses undetectable levels of PRMT6 protein due to a dearth of *PRMT6* mRNA.

When we transfected A549 cells to express Tat-FLAG (250 ng of plasmid) with or without Myc-PRMT6 (250 ng of plasmid), we observed a strong increase in Tat-FLAG protein steady-state levels in the presence of Myc-PRMT6 (Figure 4C). We have previously demonstrated that catalytically-active PRMT6 can increase the protein half-life of Tat in HeLa cells in a manner dependent on arginine methylation [13]. We therefore aimed to determine if a similar phenomenon was observable in A549 cells, which would indicate that ectopically-expressed PRMT6 is biologically active in the A549 cell line. Cells transfected to express Tat-FLAG (1 μg of plasmid) with or without coexpressing Myc-PRMT6 (1 μg of plasmid) were treated with cycloheximide (CHX) in order to arrest protein translation. At various time points post-treatment, transfected cells were harvested and assayed by western blot for Tat-FLAG, Myc-PRMT6 and endogenous β-tubulin expression. As similarly observed in Figure 4C, co expression of Myc-PRMT6 greatly enhanced the steady-state levels of Tat-FLAG just prior to CHX treatment (0 h time point), levels that were sustained by Myc-PRMT6 over the time course (Figure 4D). In contrast, Tat-FLAG levels in the absence of Myc-PRMT6 co expression quickly reduced to undetectable levels (Figure 4D). Endogenous β-tubulin protein levels remained stable throughout the time course. A plot of the Tat-FLAG band intensities over time enables calculation of Tat-FLAG protein half-lives in either the presence or absence of Myc-PRMT6 [13]. Such a calculation revealed that Myc-PRMT6 increased the protein half-life of Tat-FLAG by 5.6-fold (from 3.5 h to 19.5 h; Figure 4E). This suggested that ectopically-expressed PRMT6 can robustly increase Tat protein stability in A549 cells, thereby confirming that Myc-PRMT6 is biologically effective in A549 cells.

### PRMT6 does not down regulate HIV-1 gene expression in A549 cells

**Figure 5:**
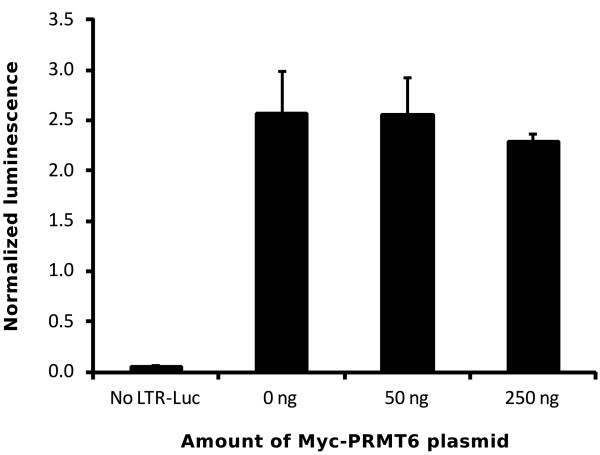
**Overexpression of Myc-PRMT6 does not affect basal transcription from the HIV-1 LTR promoter.** A549 cells were transfected with a long terminal repeat (LTR)-*Photinus* luciferase transcriptional reporter plasmid with varying amounts of Myc-PRMT6 plasmid, as indicated. All samples were cotransfected with a SV40-promotered *Renilla* luciferase plasmid to control for transfection efficiency variations. Transfected cells were lysed and assayed for both *Photinus* and *Renilla* luciferase activities, the ratio of which is shown for each sample. Columns represent the means and standard deviations of three independent experiments.

We used the A549 cell line to confirm the hypothesis that overexpression of PRMT6 down regulates Tat transactivation function. We firstly overexpressed Myc-PRMT6 in A549 cells cotransfected with a *Photinus* luciferase reporter plasmid containing the long terminal repeat (LTR), the HIV-1 promoter. This allowed us to test the effect of PRMT6 on basal transcription. When normalized for constitutive *Renilla* luciferase expressed from a SV40 promoter, we observed no change in basal transcription from the LTR in the presence of either 50 ng (*p* = 0.96, two-tailed Student’s *t* test) or 250 ng of Myc-PRMT6 plasmid (*p* = 0.30; two-tailed Student’s *t* test) compared to the no Myc-PRMT6 control (Figure 5). Next we tested the effect of PRMT6 on Tat-mediated transactivation of the LTR. Previous studies have employed HEK293T (human embryonic kidney) [7] or HeLa cells [12] to test this hypothesis, using 100 ng of Tat expression plasmid along with 0.5 μg or 1 μg of PRMT6 expression plasmid [7] or between 0.05 μg and 0.5 μg of PRMT6 plasmid [12]. In contrast, we transfected A549 cells with 0.15 ng and 0.5 ng of Tat-FLAG expression plasmid either with or without 50 ng of the wild type Myc-PRMT6 plasmid. This represents either a 333-fold or 100-fold molar excess of Myc-PRMT6 plasmid compared to Tat-FLAG plasmid, respectively. It is important to note that addition of an amino-terminal Myc tag to PRMT6 does not affect its functions [7,12,13,19,20,30,31]. We cotransfected cells with the LTR-*Photinus* luciferase reporter to indicate the effects of PRMT6 on Tat transactivation, and the SV40 promoter-*Renilla* luciferase plasmid to control for variations in transfection efficiencies and to gauge non-specific effects on plasmid expression.

**Figure 6:**
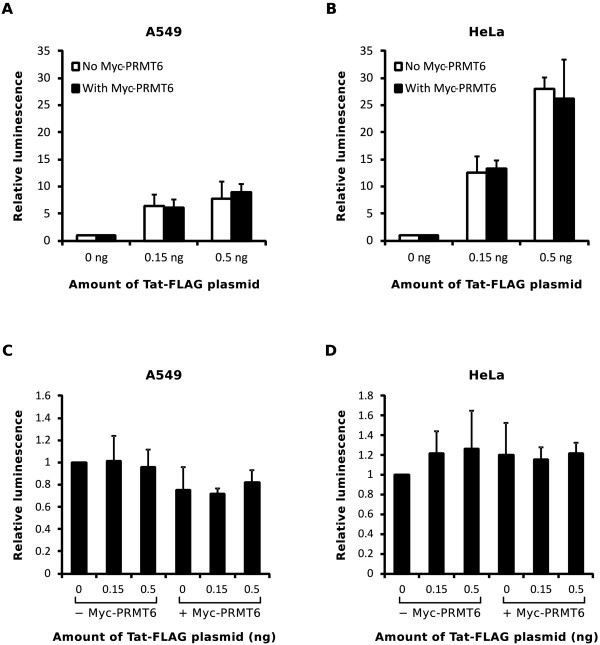
**Overexpression of Myc-PRMT6 does not alter Tat-FLAG mediated transactivation in either A549 or HeLa cells.** (**A**) A549 cells were transfected with a long terminal repeat (LTR)-*Photinus* luciferase transactivation reporter plasmid along with varying amounts of Tat-FLAG plasmid, as indicated, and either with (black columns) or without (white columns) Myc-PRMT6 plasmid. All samples were cotransfected with a SV40-promotered *Renilla* luciferase plasmid to control for transfection efficiency and assay variations. Transfected cells were lysed and assayed for both *Photinus* and *Renilla* luciferase activities, the ratio of which is shown for each sample expressed relative to the “0 ng Tat-FLAG” sample. Columns represent the means and standard deviations of four independent experiments. (**B**) The same experiment as in **A** was performed in HeLa cells. Columns represent the means and standard deviations of three independent experiments. (**C**) Overexpression of PRMT6 does not alter overall plasmid expression. The *Renilla* luciferase activities from **A** were normalized to total lysate protein concentrations before being expressed relative to the “0 ng Tat-FLAG without Myc-PRMT6” sample. Samples in which Myc-PRMT6 plasmid was (+) or was not (-) cotransfected are indicated. Columns represent the means and standard deviations of four independent experiments. (**D**) The same determinations as in **C** were performed with the samples from **B**. Columns represent the means and standard deviations of three independent experiments.

When normalized for *Renilla* luciferase expression, we saw no evidence in A549 cells of PRMT6-mediated down regulation of Tat transactivation with either 0.15 ng of Tat-FLAG plasmid (*p* = 0.39, one-tailed Student’s *t* test) or 0.5 ng of Tat-FLAG plasmid (*p* = 0.27, one-tailed Student’s *t* test; Figure 6A). To discount cell line-specific influences on the results, we repeated the experiment in HeLa cells. We again observed no PRMT6-mediated down regulation of normalized transactivation in HeLa cells with either 0.15 ng or 0.5 ng of Tat-FLAG plasmid (*p* = 0.35 and *p* = 0.34, respectively, one-tailed Student’s *t* test; Figure 6B). Importantly, there were no statistically significant differences in total-protein normalized *Renilla* luciferase expression between samples in either the A549 (Figure 6C) or HeLa (Figure 6D) cell lines (*p* > 0.05, two-tailed Welch’s *t* test), indicating that the various plasmids transfected in each experiment did not adversely affect overall expression levels. We therefore found, in contrast to previously published results [12], that PRMT6 has no statistically significant effects on Tat-mediated transactivation in either A549 or HeLa cells.

### HIV-1 produced in A549 cells overexpressing PRMT6 are competent for infection

The prevailing hypothesis is that PRMT6 is a negative regulator of HIV-1 replication, targeting not only Tat [7,12], but also the HIV-1 Rev [19] and nucleocapsid proteins [20]. We wanted to determine if PRMT6 overexpression will affect HIV-1 infectivity in our A549 cell model. A549 cells were transfected with either empty vector, wild type Myc-PRMT6 plasmid or a plasmid expressing a mutant of Myc-PRMT6 whose V86K and D88A amino acid substitutions render it catalytically inactive [7]. These cells were cotransfected with pGCH/EGFP, a proviral expression plasmid which produces HIV-1 from a CMV immediate-early promoter [13] and which contains the *EGFP* gene within the *nef* coding region of the genome. The cells were also cotransfected with a VSV-G plasmid to enable pseudotyping of virus particles. Expression of EGFP in target cells inoculated with this pseudotyped virus indicates productive infection.

**Figure 7:**
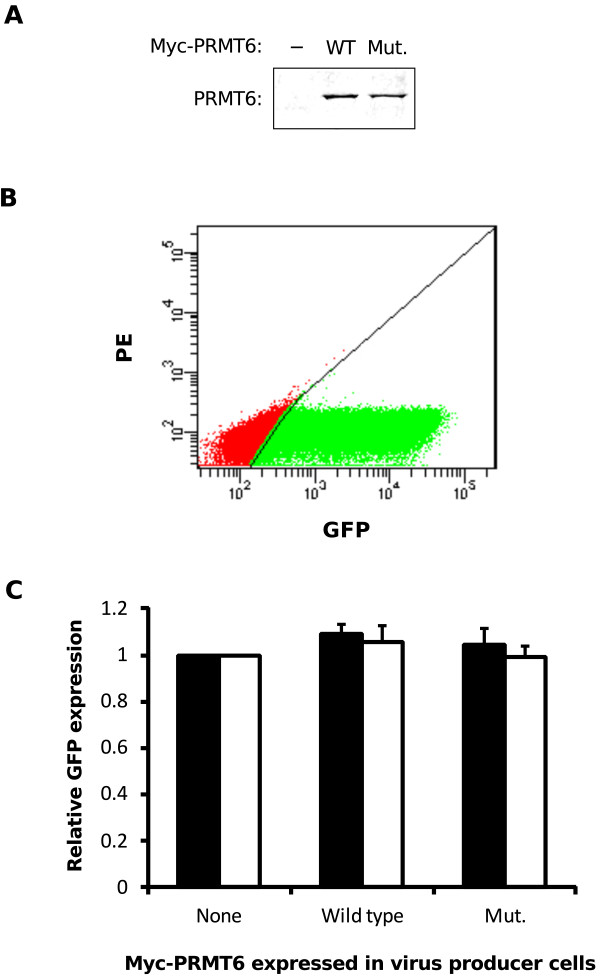
**HIV-1 produced in A549 cells overexpressing Myc-PRMT6 are competent for infectivity.** (**A**) A549 cells were transfected with a HIV-1 proviral plasmid in which the *EGFP* gene had been inserted into the *nef* reading frame, along with a plasmid expressing the VSV-G envelope glycoprotein to enable pseudotyping. Cells were simultaneously cotransfected with either empty vector (-), the wild type Myc-PRMT6 plasmid (WT) or a methyltransferase-inactive mutant Myc-PRMT6 plasmid (Mut.). Western blotting was performed on producer cell lysates using an anti-PRMT6 antibody. Loading of cell lysates was normalized for equal amounts of co expressed *Renilla* luciferase in each sample. (**B**) HeLa and A549 target cells were infected with the pseudotyped viruses collected from the A549 producer cells in **A** after normalizing for capsid levels. EGFP expression in the target cells, which indicates successful infection, was then quantified by flow cytometry. A representative dot plot of target cells infected with virus from the empty vector-cotransfected producer cells is shown, in which GFP-positive, phycoerythrin (PE)-negative cells are gated (green). (**C**) The proportions of GFP-positive HeLa (black columns) and A549 (white columns) target cells were quantified for each infection sample and are shown arranged by the Myc-PRMT6 plasmid cotransfected in the virus producer cells. Data are expressed relative to the respective empty vector sample (“None”), and columns represent the means and standard deviations of two independent infections using independent virus stocks.

After confirming expression of Myc-PRMT6 in the A549 virus producer cells (Figure 7A), both HeLa and A549 cells were exposed to equal amounts of virus collected from the producer cells and were subsequently quantified for EGFP expression by flow cytometry (Figure 7B). When expressed relative to the empty vector control, we saw no difference in EGFP expression between the HeLa cells infected with virus from the wild type Myc-PRMT6-expressing producer cells and virus from the mutant Myc-PRMT6-expressing producer cells (*p* = 0.52, two-tailed Student’s *t* test; Figure 7C). Similar results were observed when A549 cells were used as target cells (*p* = 0.39, two-tailed Student’s *t* test; Figure 7C). We therefore infer from the data that overexpression of Myc-PRMT6 in A549 virus producer cells has no impact on the subsequent infectivity of virus thus produced.

## Conclusions

### The interaction between HIV-1 Tat and PRMT6

PRMT6 was described by Boulanger and co-workers as a negative regulator of HIV-1 Tat transactivation [7]. The mechanism of action they proposed involves PRMT6-catalyzed methylation of Tat within its basic domain, which subsequently disrupts formation of the Tat-TAR-cyclin T1 ternary structure critically required for activating transcription at the HIV-1 proviral promoter [7,12]. Here we provide further insight into this proposed mechanism by demonstrating that the amino and carboxyl termini of PRMT6 interact with the activation domain of Tat. We show that the catalytic domain of PRMT6, while required for Tat methylation [7] and Tat stabilization in cells [13], is not required for interacting with Tat (Figure 1). We also demonstrate that the basic domain of Tat is dispensable for the interaction with PRMT6 (Figure 2) despite being the target domain of PRMT6-mediated methylation [7,13]. Instead, it is the amino terminal of the Tat activation domain that is essential for binding to PRMT6 (Figure 3). However, the fact that the Tat-FLAG EDE mutant could not interact with Myc-PRMT6 (Figure 3B), but the Tat-FLAG Δ16 mutant could interact (Figure 2B), suggests there may be a weaker interaction between the Tat basic domain and PRMT6 (most likely during the catalysis of Tat methylation) that is sterically hidden by the Tat activation domain.

How both the amino and carboxyl ends of PRMT6 interact with Tat is currently unclear. Finding the solution to this problem, however, is confounded by a lack of crystal structure data for PRMT6. Nonetheless, a crystal structure has been resolved for rodent PRMT1 [32], a highly conserved methyltransferase from the same family as PRMT6. The structural information for PRMT1 suggests that the amino and carboxyl termini are in opposition to each other on the same plane of the molecule [32]. If the same arrangement is true for human PRMT6, then it is plausible that both termini can simultaneously interact with Tat. This would ascribe novel roles to these regions of PRMT6, which have hitherto been functionally undefined.

The requirement for the Tat activation domain to mediate the interaction between Tat and PRMT6 is an intriguing discovery. The activation domain of Tat is critical for binding to cyclin T1 as the Tat-TAR-cyclin T1 complex assembles [1,33]. It is conceivable that binding of PRMT6 to the Tat activation domain may occlude subsequent interactions between Tat and cyclin T1. As the interaction between Tat and PRMT6 does not appear to critically require the Tat basic domain (Figure 2), this occlusion may occur either before or after the basic domain binds to TAR during the initial phases of transactivation. Recently, Sobhian and colleagues have shown that Tat can assemble functionally distinct complexes in cells, one which is transcriptionally active during HIV-1 transactivation (Tatcom1) and another which increases the available pool of the P-TEFb transcription factor complex (Tatcom2) [34]. Both of these assemblages require the binding of Tat to cyclin T1 [34]. It is possible that PRMT6 may limit the formation of such assemblages when bound to the Tat activation domain. However, our data suggest this to be unlikely. As neither basal transcription (Figure 5) nor Tat-mediated gene expression (Figure 6) were inhibited by the overexpression of PRMT6, it is likely that formation of a Tat-pTEFb complex is more favorable over a Tat-PRMT6 interaction. Further experiments, including protein binding competition assays, are required to fully explore this hypothesis.

### The A549 cell line as a model for PRMT6 function

The role of PRMT6 in HIV-1 biology is worthy of investigation, as at least three viral proteins have been demonstrated to be directly methylated by this single methyltransferase [7,19,20]. The need to develop new investigative tools and models for PRMT6 function is therefore warranted. We show here that the A549 human alveolar adenocarcinoma cell line naturally expresses undetectable levels of PRMT6 protein (Figure 4A). The absence of PRMT6 detection was attributed to the expression of low levels of *PRMT6* mRNA in the cell line (Figure 4B), corroborating data inferred from the UniGene EST profile database (Table 1) [26]. We therefore believe that the A549 cell line can be used as a model for studying the activities of PRMT6 on not only HIV, but also on other pathogens [35] and on tumorigenesis [36].

Our experiments indicated that Myc-PRMT6 increases steady-state levels of Tat-FLAG in A549 cells (Figure 4C), and that catalytically-active Myc-PRMT6 increases the protein half-life of Tat-FLAG (Figure 4D and 4E), as previously observed in HeLa cells [13]. Construing these data as evidence for biological activity of ectopic PRMT6 in A549 cells, we therefore used the A549 cell line to test two aspects of the role of PRMT6 in HIV-1 replication. Firstly, we tested the ability of PRMT6 to down regulate Tat transactivation function [7,12]. Surprisingly, we discovered that ectopically expressing PRMT6 in A549 cells (in the form of catalytically active Myc-PRMT6) had no statistically significant impact on Tat transactivation function using an LTR-luciferase reporter (Figure 6A). Importantly, we used very low amounts of Tat expression plasmid in the experiments such that the molar ratio of Myc-PRMT6 to Tat-FLAG was up to 333-fold, an amount with which any effects of PRMT6 on Tat function should have been readily observable. When we repeated the experiments in HeLa cells, in which a negative effect of PRMT6 on Tat function has been previously observed [12], we again saw no statistically significant decrease in Tat transactivation despite the 333-fold molar excess of Myc-PRMT6 to Tat-FLAG (Figure 6B).

It is unclear why differing results were observed between this study and that of Xie and colleagues [12]. One explanation could be the amount of PRMT6 expression plasmid used in the experiments. While we transfected 50 ng of PRMT6 plasmid per 2-cm^2^ well in our experiments, Xie and colleagues transfected up to 0.5 μg of PRMT6 plasmid per 3.8-cm^2^ well. Endogenous PRMT6 is known to suppress gene transcription by regulating histone post-translational modifications [15-18]. It is possible that ectopic PRMT6 may do the same, so it is therefore important to control for changes in plasmid transcription rates in experiments involving the transfection of PRMT6 expression plasmids. We observed no statistically significant negative effects on transcription in our experiments as determined with a SV40-promotered *Renilla* luciferase transfection control (Figure 6C and D). Unfortunately, the impact of PRMT6 transfection is unclear in the experiments of Xie and co-workers. Another point of difference is the clone of Tat used in the respective studies. While we used 101-amino acid Tat derived from the BH10 clone of HIV-1 (GenBank, accession number M15654), Xie and colleagues used 101-amino acid Tat derived from the HXB2 clone [37-39]. The amino acid sequences of these two clones differ in the auxiliary and second exon domains. It is possible that clonal differences of the Tat protein may account for the observed discrepancies between the two studies.

Our second test in the A549 cell line model was to assess the effect of PRMT6 on HIV-1 infectivity. This approach models the impact of PRMT6 on the viral Tat, Rev and nucleocapsid proteins, all of which play important roles during the late phase of the HIV-1 life cycle. We observed no differences in infectivity of HIV-1 produced in A549 cells cotransfected to express either an empty vector, wild type Myc-PRMT6 or a catalytically-inactive mutant of Myc-PRMT6 (Figure 7). In addition, virus levels in the culture supernatants were similar in all experiments (data not shown) suggesting that Rev, which is required for structural protein synthesis, was not inhibited. This contrasts with a study by Xie and colleagues, where HIV-1 produced in HEK293T cells in which PRMT6 was knocked down (by constitutively-expressed siRNA) was found to replicate faster in target cells within which PRMT6 was also constitutively knocked down [12]. The target cells chosen in that study were of the Jurkat lymphoblastoid cell line, a biologically more relevant target compared to the HeLa cells used in this study (Figure 7). However, it is important to note that the molecular details of PRMT6’s impact on HIV-1 have all been elucidated in HeLa or similar tissue culture lines [12,13,19,20]. Furthermore, long-term stable knock down of PRMT6 by RNA interference has an unknown effect on general cellular gene expression, especially on those genes whose expression is controlled by PRMT6-mediated methylation of histones [16,17,31,36]. It is therefore difficult to distinguish between direct (via viral proteins) and indirect (via cellular gene expression) effects of PRMT6 knock down on virus replication.

The data presented in this study prompt a revisit of the hypothesis that PRMT6 is an anti-HIV restriction factor. There is good evidence that PRMT6 can negatively influence HIV-1 replication [12], but neither its degree nor its directness is completely known. In A549 cells, a cell line that naturally expresses undetectable amounts of PRMT6 protein, there is little impact of PRMT6 overexpression on Tat-mediated transactivation or viral infectivity. In contrast, in cells that robustly express PRMT6, within which PRMT6 is constitutively knocked down by siRNA, there appears to be a significant impact on HIV-1 replication [12]. Further work is needed to determine which has the greater influence on HIV-1: the direct interactions between PRMT6 and viral proteins or the regulation of cellular gene expression by PRMT6. While PRMT6 may influence the pathogenic effects of Tat [40,41], we believe that further evidence is required before PRMT6 can firmly be considered a direct HIV-1 restriction factor.

## Methods

### Cell culture and transfections

The HeLa cell line was obtained from the ATCC biological resource center (ATCC number CCL-2). The A549 alveolar adenocarcinoma cell line [27] and the BJAB human lymphoblastoid cell line [28] were gifts from Prof. Andreas Suhrbier (Queensland Institute of Medical Research, Brisbane, Australia). All cells were cultured in RPMI 1640 medium supplemented with 100 U · ml^-1^ penicillin, 100 mg · ml^-1^ streptomycin and 10% [vol/vol] fetal bovine serum (Sigma-Aldrich Corporation), and incubated at 37°C under a humidified atmosphere of 5% CO_2_ in air. Transfections were performed with X-tremeGENE HP transfection reagent (Roche Diagnostics Corporation) according to the manufacturer’s instructions. Transfections were performed in 6-cm dishes (28 cm^2^ dish surface area) for immunoprecipitation and protein translation arrest experiments, in 6-well plates (9.6 cm^2^ well surface area) for western blotting, and in 24-well plates (2 cm^2^ well surface area) for transactivation assays.

### Plasmids

The plasmid expressing the two-exon, 101 amino-acid, BH10 clone of Tat fused to the FLAG epitope (pcDNA3.1/Tat-FLAG) was a gift from Monsef Benkirane, Institut de Génétique Humaine, Montpellier, France. The plasmids expressing fusions between the Myc epitope tag and wild type PRMT6 (pMyc-PRMT6) or methyltransferase-deficient mutant PRMT6 (pMyc-PRMT6 mut) were gifts from Stéphane Richard, McGill University, Montréal, Canada. The mutant contains V86K and D88A amino acid mutations compared to wild type PRMT6 (GenBank, accession number BC073866). The Myc-PRMT6 domain deletion mutants were created by inverse PCR using primers as described in Table 2 and using pMyc-PRMT6 as template. The Tat-FLAG domain deletion and substitution mutants were similarly created by inverse PCR using primers described in Table 2 and using pcDNA3.1/Tat-FLAG as template. Creation of the NB mutant of Tat-FLAG has been described elsewhere [42].

**Table 2:** Oligonucleotide sequences of inverse PCR primers used to construct Myc PRMT6 and Tat-FLAG mutants

**Wild type**	**Mutant**	**Forward primer**^**a**^	**Reverse primer**^**a**^
Myc-PRMT6	∆N	acggtactggacgtggg	cgaattcccgttgttcag
	∆CD	gcctccgccgagctcttc	cttgcctcgcagtgctg
	∆C1	cagcgctttgctcagcta	cggcaggagaagaccgc
	∆C2	gcgctcctctacctgaac	tggacgagccagcacgt
	∆C3	tgagaattcctgcagatatccag	ctgtttccagtgagtggcc
Tat-FLAG	∆16	cagcctaaaactgcttgtacc	catggtggcaagcttaagt
	∆CC	aggaagaagcggagacag	agcagttttaggctgacttc
	∆B	cctcctcaaggcagtcagac	gccataggagatgcctaagg
	∆SE	ggaggcgattataaggacga	ttgctttgatagagaaactt
	EDE	tcctagactagcgccctg	gctactggcgccatgg
	CS	tcttcctttcattcccaagtttct	ctttttagaataggaattggtagaagca

^a^Sequences are written 5′ to 3′.

The Tat transactivation *Photinus* luciferase reporter pGL3-LTR consists of the long terminal repeat from HIV-1 clone SF2 inserted into pGL3-basic (Promega Corporation) via *Bam* HI and *Hind* III restriction enzyme sites. The LTR spans nucleotides -180 to +81, relative to the start of transcription. The *Renilla* luciferase plasmid pRL-SV40 (Promega) was used as a transfection control in experiments where indicated. pGCH/EGFP was derived from the pGCH proviral vector [13]. Firstly, the 3′ LTR region containing the *nef* open reading frame was subcloned from pGCH to pUC19 via *Bam* HI and *Xba* I restriction sites. Secondly, the *nef* start codon was mutated to an *Age* I restriction site by inverse PCR before the *EGFP* gene (Clontech Laboratories) was inserted via the *Age* I restriction site and a pre-existing *Xho* I restriction site. Finally, the *EGFP*-containing 3′ LTR was subcloned back into pGCH via *Bam* HI and *Xba* I restriction sites. pCMV/VSV-G, which expresses the vesicular stomatitis virus envelope glycoprotein, was a kind gift from Assoc. Prof. Ian Mackay (The University of Queensland, Brisbane, Australia).

### Immunoprecipitations and western blot

For the immunoprecipitation experiments, HeLa cells in 6-cm dishes were transfected with 4 μg of Myc-PRMT6 plasmid along with 2 μg of Tat-FLAG wild-type or mutant plasmids. Cells were harvested 24 h posttransfection with phosphate-buffered saline containing 0.5 mM EDTA. The cells were washed in phosphate-buffered saline and lysed in lysis buffer (50 mM Tris, pH 7.4, 150 mM NaCl, 1 mM EDTA, protease inhibitor cocktail [Roche], 0.5% [wt/vol] Triton X-100). The lysates were centrifuged at 1000  ×  g to pellet debris, after which supernatants were collected. Lysate total protein concentrations were determined by the Bradford method [43] and, where applicable, ectopically expressed *Renilla* luciferase was quantified with the BioLux Gaussia Luciferase Flex Assay Kit (New England Biolabs). The lysates were boiled in Laemmli sample buffer [44] and electrophoresed in a denaturating polyacrylamide gel (sodium dodecyl sulfate-polyacrylamide gel electrophoresis [SDS-PAGE]) under reducing conditions. Proteins were electroblotted to a polyvinylidene fluoride membrane (Millipore Corporation) using a semidry transfer system (Bio-Rad Laboratories).

Tat-FLAG and its mutants were detected with rabbit anti-DYKDDDDK antibody (Cell Signaling Technology). Myc-PRMT6 and its mutants were detected with rabbit anti-Myc-Tag clone 71D10 antibody (Cell Signaling). Endogenous PRMT6 was detected with rabbit anti-PRMT6 antibody (LifeSpan Biosciences). PRMT1 was detected with rabbit anti-PRMT1 antibody (Epitomics). β-tubulin was detected with mouse anti-β-tubulin clone 2-28-33 antibody (Sigma-Aldrich), followed by horseradish peroxidase (HRP)-conjugated goat anti-mouse antibody (Life Technologies Corporation). Rabbit antibodies were detected with HRP-conjugated goat anti-rabbit antibody (Life Technologies). Bands were visualized with Super Signal West Pico chemiluminescent substrate (Pierce Biotechnology).

### Protein translation arrest

A549 cells growing in 6-cm dishes were transfected with 1 μg of Tat-FLAG plasmid either with or without 1 μg of Myc-PRMT6 plasmid. Dishes were treated with 60 μg · ml^-1^ of cycloheximide (Sigma-Aldrich) at 24 h post-transfection. Cells were harvested and lysed at 0, 1, 2, 4, 6 and 8 h post-treatment and lysates equivalent in total protein amounts were assayed by western blot for Tat-FLAG, Myc-PRMT6 and endogenous β-tubulin expression. Visualized bands were captured with an Image Quant LAS 500 imager (GE Healthcare) and Tat-FLAG band (pixel) intensities were quantified with ImageJ (version 1.39). The protein half-life of Tat-FLAG was calculated using the formula described previously [13].

### Quantitative RT-PCR

HeLa and A549 cells grown in 10-cm dishes were washed with phosphate-buffered saline before their total RNA were extracted with TRIzol reagent (Life Technologies) as per the manufacturer’s instructions. The total RNA were reverse transcribed with random hexamers and Superscript III MMLV RT (Life Technologies) according to the manufacturer’s instructions. *PRMT1*, *PRMT6* and *GAPDH* transcripts in the cDNA were measured by quantitative PCR using Platinum SYBR Green qPCR supermix (Life Technologies) on the Rotor-Gene Q (QIAGEN). The primers used were primers f1 and r1 for *PRMT1*, primers f2 and r2 for *PRMT6*, and primers GAPDH-f and GAPDH-r for *GADPH* as described in [36]. Calculation of *PRMT1* and *PRMT6* relative expression ratios followed the method of Pfaffl, where HeLa cells were considered the “control”, A549 cells the “sample”, and the GAPDH transcript as the “reference gene” as defined therein [29].

### Transactivation assay

For the basal transcription assay, A549 cells growing in 24-well plate wells were cotransfected with 1 μg of pGL3-LTR and 100 ng of pRL-SV40, with or without 50 ng or 250 ng of pMyc-PRMT6. For the Tat-mediated transactivation assay, HeLa or A549 cells growing in 24-well plate wells were cotransfected with 50 ng of pGL3-LTR, 50 ng of pRL-SV40, and either 0 ng, 0.15 ng or 0.5 ng of pcDNA3.1/Tat-FLAG, with or without 50 ng of pMyc-PRMT6. Cells were harvested 24 h post-transfection using Glo Lysis Buffer (Promega) as per the manufacturer’s instructions. *Photinus* luciferase was quantified with the Steady-Glo Luciferase Assay System (Promega), and *Renilla* luciferase was quantified with the BioLux Gaussia Luciferase Flex Assay Kit (New England Biolabs), as per the manufacturers’ instructions. Total protein concentrations in lysates were quantified by the Bradford method [43].

### Virus production, infection and flow cytometry

A549 cells in 10-cm dishes were transfected with 5 μg of pGCH/EGFP, 2.5 μg of pCMV/VSV-G and 2.5 μg of either pcDNA3.1 (“empty vector”), pMyc-PRMT6 or pMyc-PRMT6 mut. Virus particles in the culture media were collected 48 h post-transfection and quantified for viral capsid levels by ELISA (Zeptometrix Corporation). HeLa cells growing in 6-well plates at 2  ×  10^5^ cells per well were infected with 20 ng per well (capsid equivalent) of virus in the presence of 8 μg · ml^-1^ hexadimethrine bromide. Cells were harvested 42 h post-infection with phosphate-buffered saline containing 0.5 mM EDTA (EDTA/PBS) before being fixed with 0.5% [wt/vol] formaldehyde for 5 min. Cells were washed and resuspended in EDTA/PBS in order to analyze EGFP expression with a LSR Fortessa flow cytometer (Becton, Dickinson and Company). Cells were gated on side-scatter and forward-scatter parameters and excited for fluorescence with the 488 nm and 561 nm light sources.

### Statistical analyses

Hartley’s test was used to determine homoscedasticity (equivalence of variance) between data sets. Student’s *t* test was used to evaluate null hypotheses for homoscedastic data, while Welch’s *t* test was used for heteroscedastic data. Rejection of null hypotheses occurred when *p* < 0.05.

## Abbreviations

PRMT6: Protein arginine methyltransferase 6; CHX: Cycloheximide; LTR: Long terminal repeat; TAR: Transactivation response element.

## Competing interests

The authors declare that they have no competing interests.

## Authors' contributions

HS and ML made substantial contributions to conception and design of the experiments, acquisition of data, analysis and interpretation of the data, and have been involved in the drafting of the manuscript. AA, HJ, DL and TW provided critical feedback on experimental design and critically evaluated the manuscript for important intellectual content. DH made substantial contributions to conception and design of the experiments, analysis and interpretation of the data, has been involved in the drafting of the manuscript, provided critical feedback on experimental design, and critically evaluated the manuscript for important intellectual content. All authors read and approved the final manuscript.

## References

[B1] Wei P Garber ME Fang SM Fischer WH Jones KA A novel CDK9-associated C-type cyclin interacts directly with HIV-1 tat and mediates its high-affinity, loop-specific binding to TAR RNA Cell 1998 92 451 462 10.1016/S0092-8674(00)80939-3 9491887

[B2] Yang Z Zhu Q Luo K Zhou Q The 7SK small nuclear RNA inhibits the CDK9/cyclin T1 kinase to control transcription Nature 2001 414 317 322 10.1038/35104575 11713532

[B3] Barboric M Lenasi T Kick-sTARting HIV-1 transcription elongation by 7SK snRNP deporTATion Nat Struct Mol Biol 2010 17 928 930 10.1038/nsmb0810-928 20683478

[B4] D'Orso I Frankel AD RNA-mediated displacement of an inhibitory snRNP complex activates transcription elongation Nat Struct Mol Biol 2010 17 815 821 10.1038/nsmb.1827 20562857 PMC2921552

[B5] Hetzer C Bisgrove D Cohen MS Pedal A Kaehlcke K Speyerer A Bartscherer K Taunton J Ott M Recruitment and activation of RSK2 by HIV-1 Tat PLoS One 2007 2 e151 10.1371/journal.pone.0000151 17225856 PMC1764712

[B6] Yedavalli VR Jeang KT Methylation: a regulator of HIV-1 replication? Retrovirology 2007 4 9 10.1186/1742-4690-4-9 17274823 PMC1796896

[B7] Boulanger MC Liang C Russell RS Lin R Bedford MT Wainberg MA Richard S Methylation of tat by PRMT6 regulates human immunodeficiency virus type 1 gene expression J Virol 2005 79 124 131 10.1128/JVI.79.1.124-131.2005 15596808 PMC538702

[B8] Van Duyne R Easley R Wu W Berro R Pedati C Klase Z Kehn-Hall K Flynn EK Symer DE Kashanchi F Lysine methylation of HIV-1 tat regulates transcriptional activity of the viral LTR Retrovirology 2008 5 40 10.1186/1742-4690-5-40 18498648 PMC2412914

[B9] Pagans S Kauder SE Kaehlcke K Sakane N Schroeder S Dormeyer W Trievel RC Verdin E Schnolzer M Ott M The Cellular lysine methyltransferase Set7/9-KMT7 binds HIV-1 TAR RNA, monomethylates the viral transactivator tat, and enhances HIV transcription Cell Host Microbe 2010 7 234 244 10.1016/j.chom.2010.02.005 20227666 PMC2844784

[B10] Sakane N Kwon HS Pagans S Kaehlcke K Mizusawa Y Kamada M Lassen KG Chan J Greene WC Schnoelzer M Ott M Activation of HIV transcription by the viral tat protein requires a demethylation step mediated by lysine-specific demethylase 1 (LSD1/KDM1) PLoS Pathog 2011 7 e1002184 10.1371/journal.ppat.1002184 21876670 PMC3158049

[B11] Hetzer C Dormeyer W Schnolzer M Ott M Decoding tat: the biology of HIV tat posttranslational modifications Microbes Infect 2005 7 1364 1369 10.1016/j.micinf.2005.06.003 16046164

[B12] Xie B Invernizzi CF Richard S Wainberg MA Arginine methylation of the human immunodeficiency virus type 1 tat protein by PRMT6 negatively affects tat interactions with both cyclin T1 and the tat transactivation region J Virol 2007 81 4226 4234 10.1128/JVI.01888-06 17267505 PMC1866113

[B13] Sivakumaran H van der Horst A Fulcher AJ Apolloni A Lin MH Jans DA Harrich D Arginine methylation increases the stability of human immunodeficiency virus type 1 tat J Virol 2009 83 11694 11703 10.1128/JVI.00499-09 19726520 PMC2772670

[B14] Wolf SS The protein arginine methyltransferase family: an update about function, new perspectives and the physiological role in humans Cell Mol Life Sci 2009 66 2109 2121 10.1007/s00018-009-0010-x 19300908 PMC11115746

[B15] Guccione E Bassi C Casadio F Martinato F Cesaroni M Schuchlautz H Luscher B Amati B Methylation of histone H3R2 by PRMT6 and H3K4 by an MLL complex are mutually exclusive Nature 2007 449 933 937 10.1038/nature06166 17898714

[B16] Hyllus D Stein C Schnabel K Schiltz E Imhof A Dou Y Hsieh J Bauer UM PRMT6-mediated methylation of R2 in histone H3 antagonizes H3 K4 trimethylation Genes Dev 2007 21 3369 3380 10.1101/gad.447007 18079182 PMC2113036

[B17] Iberg AN Espejo A Cheng D Kim D Michaud-Levesque J Richard S Bedford MT Arginine methylation of the histone H3 tail impedes effector binding J Biol Chem 2008 283 3006 3010 18077460 10.1074/jbc.C700192200

[B18] Kirmizis A Santos-Rosa H Penkett CJ Singer MA Vermeulen M Mann M Bahler J Green RD Kouzarides T Arginine methylation at histone H3R2 controls deposition of H3K4 trimethylation Nature 2007 449 928 932 10.1038/nature06160 17898715 PMC3350864

[B19] Invernizzi CF Xie B Richard S Wainberg MA PRMT6 diminishes HIV-1 Rev binding to and export of viral RNA Retrovirology 2006 3 93 10.1186/1742-4690-3-93 17176473 PMC1779295

[B20] Invernizzi CF Xie B Frankel FA Feldhammer M Roy BB Richard S Wainberg MA Arginine methylation of the HIV-1 nucleocapsid protein results in its diminished function AIDS 2007 21 795 805 10.1097/QAD.0b013e32803277ae 17415034

[B21] Frankel A Yadav N Lee J Branscombe TL Clarke S Bedford MT The novel human protein arginine N-methyltransferase PRMT6 is a nuclear enzyme displaying unique substrate specificity J Biol Chem 2002 277 3537 3543 10.1074/jbc.M108786200 11724789

[B22] Kuppuswamy M Subramanian T Srinivasan A Chinnadurai G Multiple functional domains of tat, the trans-activator of HIV-1, defined by mutational analysis Nucleic Acids Res 1989 17 3551 3561 10.1093/nar/17.9.3551 2542902 PMC317795

[B23] Garcia JA Harrich D Pearson L Mitsuyasu R Gaynor RB Functional domains required for tat-induced transcriptional activation of the HIV-1 long terminal repeat EMBO J 1988 7 3143 3147 3181132 10.1002/j.1460-2075.1988.tb03181.x PMC454704

[B24] Reddy MV Desai M Jeyapaul J Prasad DD Seshamma T Palmeri D Khan SA Functional analysis of the N-terminal domain of tat protein of the human immunodeficiency virus type 1 Oncogene 1992 7 1743 1748 1501886

[B25] Ruben S Perkins A Purcell R Joung K Sia R Burghoff R Haseltine WA Rosen CA Structural and functional characterization of human immunodeficiency virus tat protein J Virol 1989 63 1 8 2535718 10.1128/jvi.63.1.1-8.1989 PMC247650

[B26] Pontius JU Wagner L Schuler GD McEntyre J, Ostell J, Bethesda MD UniGene: a unified view of the transcriptome The NCBI Handbook 2003: National Center for Biotechnology Information

[B27] Giard DJ Aaronson SA Todaro GJ Arnstein P Kersey JH Dosik H Parks WP In vitro cultivation of human tumors: establishment of cell lines derived from a series of solid tumors J Natl Cancer Inst 1973 51 1417 1423 4357758 10.1093/jnci/51.5.1417

[B28] Menezes J Leibold W Klein G Clements G Establishment and characterization of an epstein-barr virus (EBC)-negative lymphoblastoid B cell line (BJA-B) from an exceptional, EBV-genome-negative African burkitt's lymphoma Biomedicine 1975 22 276 284 179629

[B29] Pfaffl MW A new mathematical model for relative quantification in real-time RT-PCR Nucleic Acids Res 2001 29 e45 10.1093/nar/29.9.e45 11328886 PMC55695

[B30] El-Andaloussi N Valovka T Toueille M Steinacher R Focke F Gehrig P Covic M Hassa PO Schar P Hubscher U Hottiger MO Arginine methylation regulates DNA polymerase beta Mol Cell 2006 22 51 62 10.1016/j.molcel.2006.02.013 16600869

[B31] Michaud-Levesque J Richard S Thrombospondin-1 is a transcriptional repression target of PRMT6 J Biol Chem 2009 284 21338 21346 10.1074/jbc.M109.005322 19509293 PMC2755858

[B32] Zhang X Cheng X Structure of the predominant protein arginine methyltransferase PRMT1 and analysis of its binding to substrate peptides Structure 2003 11 509 520 10.1016/S0969-2126(03)00071-6 12737817 PMC4030380

[B33] Zhang J Tamilarasu N Hwang S Garber ME Huq I Jones KA Rana TM HIV-1 TAR RNA enhances the interaction between tat and cyclin T1 J Biol Chem 2000 275 34314 34319 10944537 10.1074/jbc.M006804200

[B34] Sobhian B Laguette N Yatim A Nakamura M Levy Y Kiernan R Benkirane M HIV-1 tat assembles a multifunctional transcription elongation complex and stably associates with the 7SK snRNP Mol Cell 2010 38 439 451 10.1016/j.molcel.2010.04.012 20471949 PMC3595998

[B35] Dove BK Surtees R Bean TJ Munday D Wise HM Digard P Carroll MW Ajuh P Barr JN Hiscox JA A quantitative proteomic analysis of lung epithelial (A549) cells infected with 2009 pandemic influenza a virus using stable isotope labelling with amino acids in cell culture Proteomics 2012 12 1431 1436 10.1002/pmic.201100470 22585751

[B36] Yoshimatsu M Toyokawa G Hayami S Unoki M Tsunoda T Field HI Kelly JD Neal DE Maehara Y Ponder BA Dysregulation of PRMT1 and PRMT6, type I arginine methyltransferases, is involved in various types of human cancers Int J Cancer 2011 128 562 573 10.1002/ijc.25366 20473859

[B37] Ott M Emiliani S Van Lint C Herbein G Lovett J Chirmule N McCloskey T Pahwa S Verdin E Immune hyperactivation of HIV-1-infected T cells mediated by tat and the CD28 pathway Science 1997 275 1481 1485 10.1126/science.275.5305.1481 9045614

[B38] Ott M Schnolzer M Garnica J Fischle W Emiliani S Rackwitz HR Verdin E Acetylation of the HIV-1 tat protein by p300 is important for its transcriptional activity Curr Biol 1999 9 1489 1492 10.1016/S0960-9822(00)80120-7 10607594

[B39] Arya SK Guo C Josephs SF Wong-Staal F Trans-activator gene of human T-lymphotropic virus type III (HTLV-III) Science 1985 229 69 73 10.1126/science.2990040 2990040

[B40] Rayne F Debaisieux S Yezid H Lin YL Mettling C Konate K Chazal N Arold ST Pugniere M Sanchez F Phosphatidylinositol-(4,5)-bisphosphate enables efficient secretion of HIV-1 tat by infected T-cells EMBO J 2010 29 1348 1362 10.1038/emboj.2010.32 20224549 PMC2868572

[B41] Johri MK Mishra R Chhatbar C Unni SK Singh SK Tits and bits of HIV tat protein Expert Opin Biol Ther 2011 11 269 283 10.1517/14712598.2011.546339 21204735

[B42] Meredith LW Sivakumaran H Major L Suhrbier A Harrich D Potent inhibition of HIV-1 replication by a tat mutant PLoS One 2009 4 e7769 10.1371/journal.pone.0007769 19901984 PMC2768900

[B43] Bradford MM A rapid and sensitive method for the quantitation of microgram quantities of protein utilizing the principle of protein-dye binding Anal Biochem 1976 72 248 254 10.1016/0003-2697(76)90527-3 942051

[B44] King J Laemmli UK Polypeptides of the tail fibres of bacteriophage T4 J Mol Biol 1971 62 465 477 10.1016/0022-2836(71)90148-3 5136579

